# GermVersity: A free and user-friendly interface to enhance the visualization and analysis of genebank data

**DOI:** 10.1371/journal.pone.0340826

**Published:** 2026-01-12

**Authors:** Joaquin Guillermo Ramirez-Gil, Felipe López-Hernández, Diego Felipe Conejo-Rodriguez, Juan Camilo Henao-Rojas, Kevin Estiven Quiroga-Benavides, Andrés J. Cortés, Maria Isabel Chacón-Sánchez

**Affiliations:** 1 Departamento de Agronomía, Facultad de Ciencias Agrarias, Universidad Nacional de Colombia, Bogotá, Colombia; 2 Laboratorio de Agrocomputación y Análisis epidemiológico, Center of Excellence in Scientific Computing, Universidad Nacional de Colombia,; 3 Corporación Colombiana de Investigación Agropecuaria– Agrosavia, Centro de Investigación La Selva, Rionegro, Colombia; 4 Genetic Resources Program, International Center for Tropical Agriculture, Bean physiology, International Center for Tropical Agriculture, Palmira, Valle del Cauca, Colombia; 5 Facultad de Ciencias Agrarias, Departamento de Ciencias Forestales, Universidad Nacional de Colombia - Sede Medellín, Medellín, Colombia; National Bureau of Plant Genetic Resources, INDIA

## Abstract

Genebanks are crucial for food security and industrial applications. However, their heterogeneous nature hinders effective utilization. To address this, the GermVersity platform was developed to integrate conventional, artificial intelligence, and data science approaches to the transversal analysis of data associated with genebank accessions. GermVersity aids in the classification and prioritization of germplasm diversity, facilitating the conservation and utilization of valuable genetic resources in agriculture. The GermVersity proposal is focused on developing a user-friendly application to visualize, analyze and interpret analysis-ready data from genebanks. The application’s case study uses data from the bean genebank at Bioversity International – CIAT but can be adapted for other genebanks and broader collections. GermVersity is divided into three modules. The first module analyzes genetic diversity using phenotypic and SNP data. In this module users can prioritize morphological descriptors for efficient classification of accessions, define genetic clusters or populations, calculate basic diversity statistics per locus and per population, and estimate genetic divergence among clusters or populations. The second module implements spatial distribution modeling, allowing users to understand ecological diversity across the geographical distribution range of a set of accessions. The third module characterizes the genomic architecture of phenotypic trait variation and adaptation to ecological niches using genome-wide association implemented in prediction integrated tools (GAPIT) and Latent Factor Mixed Model (LFMM) analysis. The GermVersity platform was built using Shiny-Golem and is hosted on GitHub for free use. To access it, users can install the library and run the application through specific commands in R. Responsible use of algorithms is recommended, along with the inspection of results for biological coherence. Ultimately, GermVersity offers an integrated analytical pipeline to leverage genebank diversity as part of pre-breeding efforts.

## Introduction

The use and conservation of germplasm banks in agriculture represents one of the most precious assets of humanity in terms of guaranteeing food security and sovereignty [1–3]. Moreover, it is currently the basis for potential bioeconomical applications in cosmetics and other industries, for the discovery of bioactive compounds and antimicrobials, and many other uses [4]. Despite their value, highly heterogeneous collections are often difficult to characterize and utilize at the phenotypic and genetic level, particularly within pre-breeding programs of donor accessions from landrace and Crop Wild Relative (CWR) genepools. Therefore, developing new tools to better understand and leverage the hidden variation stored at germplasm banks is a prerequisite to unlock their potential.

Once discovered, genetic resources in agriculture may contribute to avoiding genetic erosion while promoting phenotypic innovation; therefore, we must guarantee their conservation and generate as much data from existing collections as possible [5,6]. Modern trends in germplasm studies include characterization of the genetic basis of phenotypic and adaptive variation, reconstruction of evolutionary, ecological and selective processes shaping CWR genepools, and genebank omics screening aiming to unveil novel value and uses for human communities [5,7]. A common denominator across these tasks is the efficient management and characterization of germplasm banks using accurate and low cost tools for data acquisition, storing and processing [7–10].

The implementation of documentation systems is essential not only for genebank management but also for an efficient use of the germplasm (revised in [11]). A large amount of data and information related to passport data, characterization, evaluation, management and distribution need to be documented in a well-structured and suitable database, which may require expert input and resources. Therefore, data management is one of the main challenges of genebanks. Of all the data maintained by genebanks, characterization and evaluation data are particularly important to classify and identify germplasm of interest for breeding purposes or other uses. Collecting evaluation data for many traits of interest may imply high costs and, for this reason, they are usually collected by genebanks during seed regeneration cycles or by external users (e.g., breeders). In view of this situation, evaluation data collected in different ways may not be comparable and their use for downstream applications may be challenging or impossible. Consequently, users should be aware that these data may not be ready for analysis and may need further processing strategies that enable their use, such as those reported by Phillip et al. [12]. Once data are ready for analysis, users need to use appropriate tools to classify and identify germplasm that is useful for their research purposes.

In this regards, artificial intelligence, data science, programming and multiple mathematical and statistical methods are emerging tools with various uses, including in agriculture and biological systems. Of these technologies, Decision Support Systems (DSS) and Internet of Things (IoT)-based platforms stand out [13,14]. Many of these DSS are based on Digital Platforms (DP) with a friendly end-user interface with the ability to manage data, visualize trends and emerging properties, and determine causality relationships in order to subsequently make informed forecasts and selections [15]. Digital platforms have commonly been used in recent years, providing multiple comparative advantages. They can offer alternatives for the analysis of data associated to germplasm collections [8,16–18]. In this regard, DP must meet a series of conditions in order to be adopted by potential users. Specifically, DP must be free, user friendly, innovative, computationally efficient, and reliable [8,19].

Here, we present GermVersity, a free software and graphical interface that was developed to help non-specialized users and researchers in the analysis and visualization of multidimensional data associated with germplasm accessions. GermVersity harbors a series of analytical tools that can be used for diverse purposes such as the classification of germplasm accessions based on phenotypic traits, prioritization of descriptors for management of a germplasm collection, identification of geographical areas and ecological gradients with potential new diversity, assessment of the genetic structure of a germplasm collection, and identification of genetic variants related to agronomic traits or environmental variables of adaptive significance. The ultimate purpose of GermVersity is to assist users in the efficient analysis and utilization of data to uncover the potential of germplasm accessions, and thus enhance their conservation and use in breeding programs.

## Materials and methods

### General description of GermVersity platform

**Fig 1:**
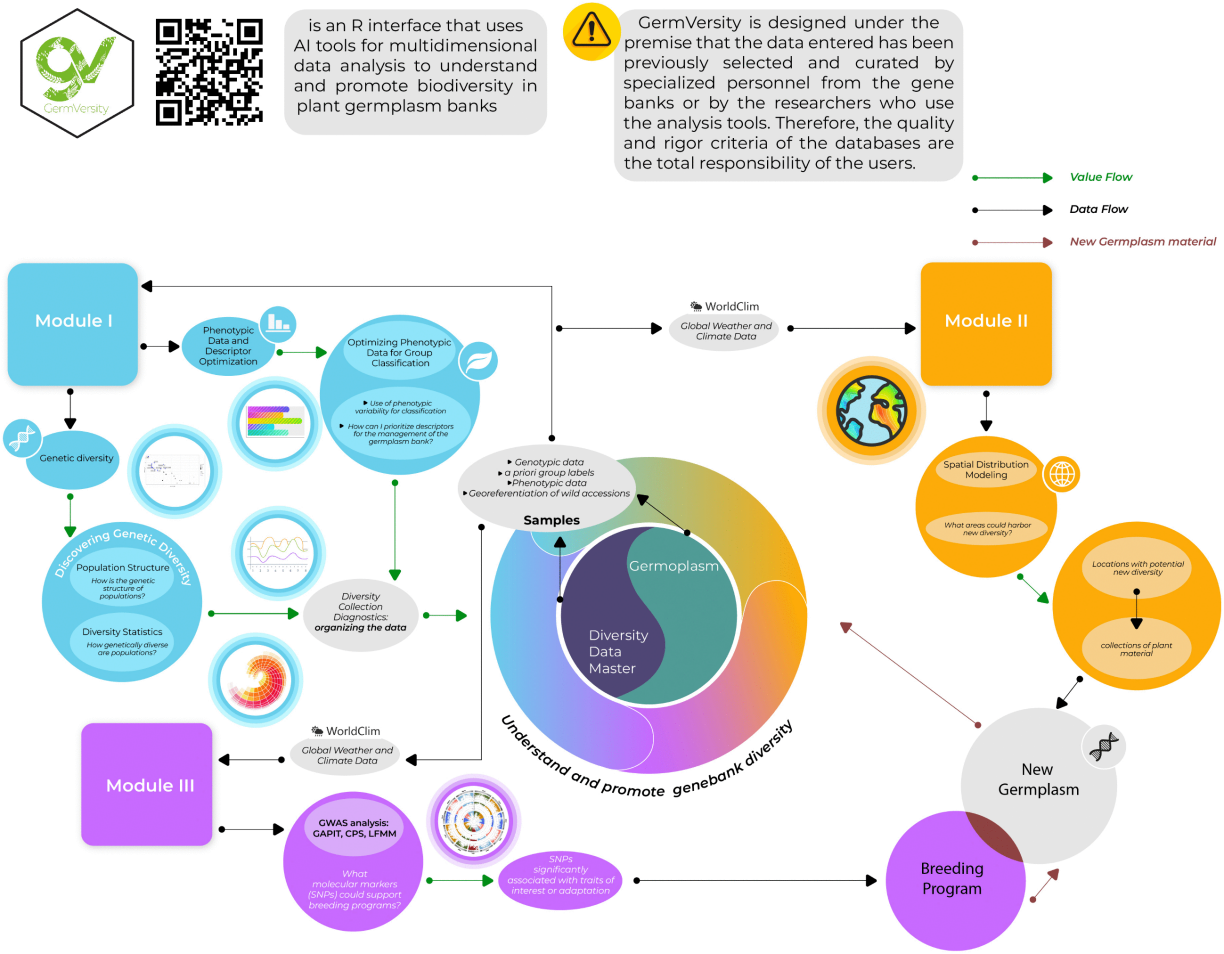
GermVersity general scheme. General scheme, data flow and value flow of the GermVersity platform, and its usability in genebank systems.

Our GermVersity proposal was based on developing an application that offers a user-friendly interface for visualizing and advancing analysis of data obtained from germplasm banks (Fig 1). In our case, we have focused on proposing a user platform that enhances understanding of phenotypic and genetic diversity, as well as spatial patterns within a geographical context. Therefore, GermVersity requires high-quality data enriched with phenotypic, genetic, and geographic characteristics to achieve the analyses we propose. Similarly, as suggested, responsible use of the platform and detailed analysis of the quality, quantity, and accuracy of the data are necessary before engaging in any exercises with the proposed platform.

For the guided case study provided to users, the analyses implemented in the three modules utilized databases from the bean germplasm bank (*Phaseolus* genus) at the International Center for Tropical Agriculture (Bioversity International – CIAT). This selection is based on the working group’s experience and access to data for this genus. This does not preclude that GermVersity can be utilized for any other gene banks and plant collections that the user wishes. Nonetheless, responsible use of the implemented algorithms is recommended, seeking to comply with the principles or theoretical assumptions, as well as inspection of the results for biological and ecological coherence.

GermVersity is designed on the premise that data inputs have been previously curated by genebank personnel or researchers employing the analyses and, as such, does not incorporate a specific curation module. In addition, for the demonstrative data showcased in our submission, we relied on databases that have been meticulously curated by experts and quality systems established by the Bioversity-CIAT alliance. Additionally, GermVersity aims to enhance the analytical capacity of data associated with germplasm banks, such as phenotypic (Module 1), geographic (Module 2), and genotypic (Module 3) characterization, where the data must be acquired, managed, and preprocessed beforehand, following strict protocols for this purpose. In this regard, GermVersity enables a final phase of advanced and informative analysis to enhance the understanding of genetic resources. As described above, GermVersity was divided into three main modules with internal sub-modules. Each submodule was associated with analysis tools for specific purposes. The details of the design and use of the platform, as well as the analyses, underlying libraries, data requirements, and suggested work flowchart are described in detail below.

To ensure the appropriate utilization of GermVersity for educational and research purposes, users are strongly encouraged to consult the comprehensive user manual, as well as the individual documentation associated with each module. The platform has been designed to be accessed either through a Shiny-based graphical interface or by executing the modular scripts in Markdown format, depending on user preference and technical expertise. For optimal performance and reproducibility, it is essential that users adhere strictly to the structure and format of the example datasets and scripts provided in the project’s official open-access repository (https://github.com/agrocompuepidemlab/GermVersity/releases/tag/v1.0.0). All resources, including manuals, codes, and datasets, are available in both English and Spanish. It is important to note that execution errors may arise from inconsistencies in data formatting or version incompatibilities within the R environment. Therefore, we recommend users carefully follow the step-by-step instructions as outlined in the repository: https://github.com/agrocompuepidemlab/GermVersity/releases/tag/v1.0.0.

### GermVersity platform design and general access instructions

GermVersity was built with Shiny-Golem version 0.3.0 (R language), which is a framework that allows the creation of R packages (R version 4.3.3). Executing a command starts a web application that will be automatically opened in a browser. The web application was built with Shiny, ShinyDashboard (version 0.7.2) and ShinydashboardPlus (version 2.0.3), which enables interaction with the user, allowing the upload of files and the display of figures resulting from the analyses carried out internally. The package contains a single function that initializes the web application. This interface allows users to easily perform multiple analyses based on the selected module and to upload databases in the format and type described in each module. Sample datasets and example files are available in the freely accessible repository where all data, codes, user manual and video are hosted (https://github.com/agrocompuepidemlab/GermVersity/releases/tag/v1.0.0).

GermVersity is hosted as a library downloadable from GitHub, where users can access instructions for installing the library on a local machine using the following command *devtools::install_github(‘GermVersity/GermVersity’)*. Therefore, the user will have complete control over their data, results, privacy, and computing capacity for their analyses based on the specifications of their personal computer. For the installation of the library, it is important to follow the instructions within the repository, since it must be guaranteed that four libraries are previously installed and compatible with the user’s current R version (*i.e.*, *Pomona*, *GAPIT3*, *LEA*, *qvalue*). Without these libraries the package will not be installed. All the R libraries and functions used by GermVersity are shown in the user manual. To facilitate accessibility, we have added alternative installation methods, including the use of BiocManager (Bioconductor) and devtools/remotes for GitHub dependencies, to complement CRAN and improve reproducibility. We have restructured the order of library loading in app-config.R to minimize potential conflicts and ensure smooth installation and updates.

**Fig 2:**
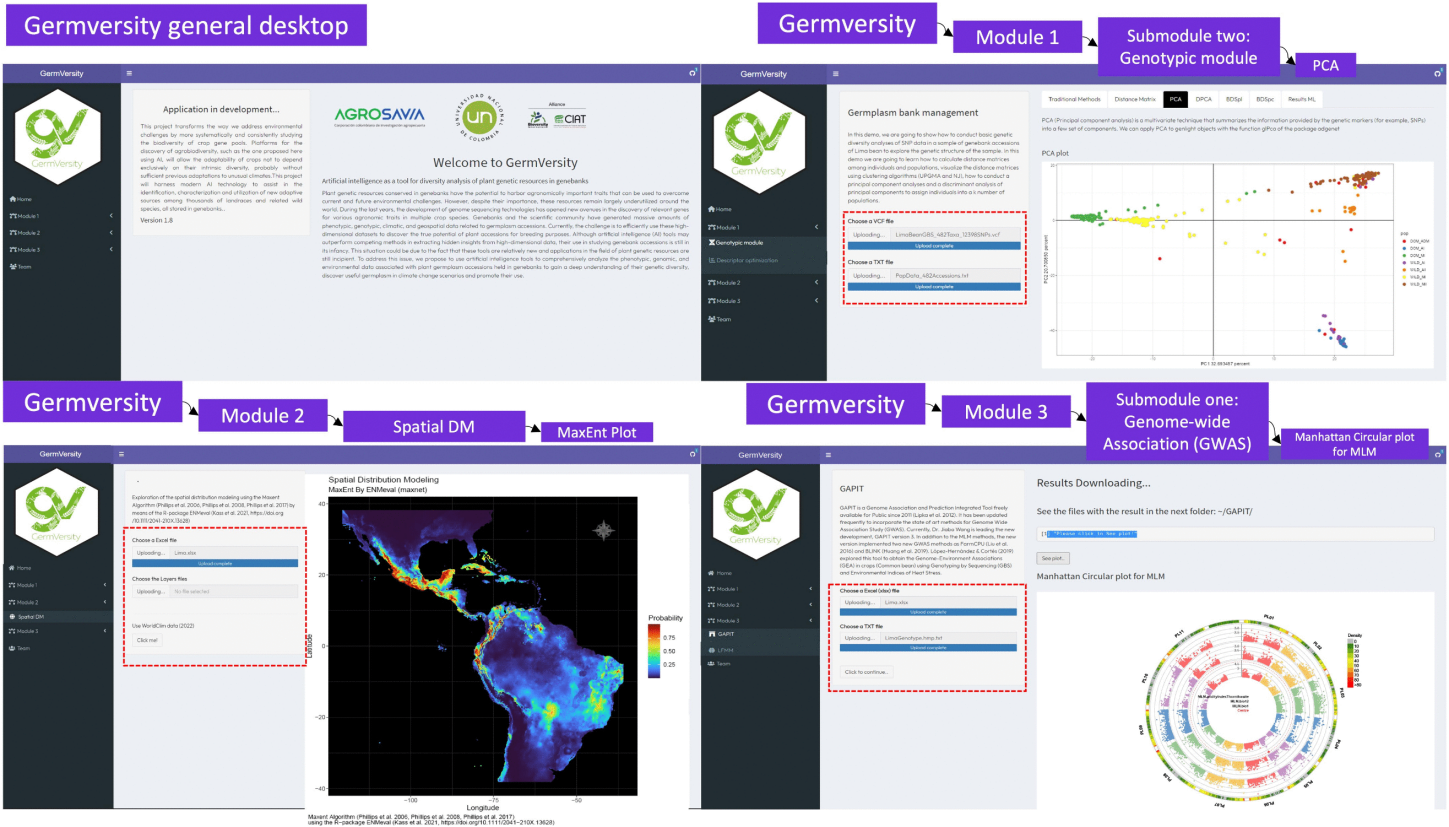
Screenshots of the GermVersity graphical interface. Real view of the GermVersity graphical interface, general desktop, and each of its three analysis modules.

Once the *GermVersity* library is installed, it is possible to run the application without having to import the library by executing the following command in R: *GermVersity::run_GermVersity()*, which will automatically display the url 127.0.0.1:4489 in the user’s browser. Once the application is executed, it will be possible to upload input files at each module. This will internally start the processing for the visualization of the tools. In Fig 2, the components of the graphical interface of GermVersity are described.

As several R packages no longer have support in CRAN, and therefore cannot be installed, the way to install these packages is through their source. To address the issue of R libraries that are no longer supported or available via CRAN, we created a local library repository that includes the necessary package versions required for the proper functioning of the platform (https://github.com/agrocompuepidemlab/GermVersity/releases/tag/v1.0.0). Specifically, packages such as maptools (v1.1-8), snpStats (v1.56.0), and LDheatmap (v1.0-6) have been included in the “Library” folder located within the main project directory (https://github.com/agrocompuepidemlab/GermVersity/releases/tag/v1.0.0). Users are advised to remain vigilant regarding package obsolescence, as updates to CRAN may render some dependencies unsupported. Installation of these packages should be performed manually through the R package installation interface. In the “Install from” section, select the option “Package Archive File (.zip;.tar.gz)”, and install the packages in the following order: first maptools, then snpStats, and finally LDheatmap. Please ensure the genetics package is installed prior to LDheatmap, as it is a required dependency.

In the official GermVersity repository (https://github.com/agrocompuepidemlab/GermVersity/releases/tag/v1.0.0) complete instructions for the installation of the packages that no longer have support as of 2025, and for the successful use the platform, can be found. Users are strongly encouraged to follow the step-by-step instructions provided in both the general and module-specific user manuals. Doing so ensures proper use of the platform, accurate execution of analyses, and avoids errors related to unsupported or outdated R libraries not currently available through CRAN.

If the user requires greater computing capacity, GermVersity can be run under other environments such as fixed servers, virtual servers without the need for hardware or software like RStudio Cloud (https://rstudio.cloud/), services such as Platform as a Service (PaaS), or other types of servers in the cloud. This allows the user to always have control over their data, processing characteristics (*e.g.,* speed), and whether they perform analyses online or offline. Similarly, if users wish, they can run the codes one by one without using the Shiny user interface by utilizing each of the shared scripts in Markdown format.

### What analytics does the GermVersity platform include?

GermVersity is divided into three modules. The first module carries out basic diversity analyses in a sample of germplasm accessions using phenotypic and SNP (Single-Nucleotide Polymorphism) data. This module is subdivided into two submodules. In submodule one, the user can identify, based on machine learning, the phenotypic descriptors that are most useful in the classification of genebank accessions and functional groups. The user can also explore the phenotypic diversity in a sample of accessions by means of a principal component analysis, *K*-means clustering and hierarchical clustering analysis. In submodule two, the genetic stratification of a sample of germplasm accessions, genotyped at SNP markers, can be determined and individuals can be assigned to populations with diverse tools such as clustering analysis, Principal Component Analysis (PCA) and Discriminant Analysis of Principal Components (DAPC). Also, basic diversity statistics (genome-wide and per SNP locus) can be computed for the whole sample and for each cluster, as well as estimates of global genetic divergence among all clusters or among pairs of clusters. The second module is devoted to Spatial Distribution Modeling (SDM) aiming to comprehend ecological diversity and standing adaptation at germplasm collections, taking into account the wide range of niche gradients. This module offers two approaches to SDM, a classical function using a Generalized Linear Model (GLM) and the extensively used Maximum Entropy (MaxEnt) approach. The third module aims to characterize the genomic architecture of relevant phenotypic and adaptive trait variation in germplasm collections. It is divided into two submodules: Genome Association using Prediction Integrated Tool (GAPIT) and Latent Factor Mixed Model (LFMM) analysis. The GAPIT submodule utilizes FarmCPU (Fixed and random model Circulating Probability Unification), BLINK (Bayesian-information and Linkage-disequilibrium Iteratively Nested Keyway) and MLM (Mixed Linear Model) algorithms. The second submodule computes a LFMM analysis to calculate alternative correlations between genetic and environmental variables.

Throughout the text, the letter *K* is used to refer to the number of clusters or populations, defined by *K*-means clustering or other methods, and on the basis of phenotypic variables or molecular markers. *K* may also refer to the number of latent factors (or unobserved confounders) used in LFMM.

### Data requirements and specific description of analyses included in GermVersity

#### Module one: Optimization and determination of relevance of descriptors and analysis of the phenotypic and genotypic diversity of genebanks.

As stated before, genebanks are not only responsible for collecting and maintaining germplasm collections, but also for documenting and characterizing the collections in order to enhance their use. In many cases, genebanks not only register basic passport data for each accession, but also collect a series of trait data (*i.e.*, morpho-agronomic data) in multiple sites or years to describe the accessions. An accurate evaluation of morpho-agronomic traits is labor-intensive and, in numerous cases, these valuable data are not further used for measuring the genetic diversity of the collection or for breeding purposes. In other cases, genebanks also collect molecular marker data, which may provide valuable and complementary information. A comprehensive analysis of the genetic diversity held in a genebank could offer useful information for breeders. For example, deciphering patterns of genetic structure and linkage disequilibrium, with selected descriptors and molecular marker data, is key when carrying out robust Genome-Wide Association Studies (GWAS) and in the identification of Quantitative Trait Loci (QTL) that can be used in Marker Assisted Selection (MAS). Also, genetic diversity analyses are necessary to establish core collections, which minimize the number of accessions that breeders need to screen for useful traits. For all these reasons, this module implements methods based on machine learning or on traditional approaches with the aim to assist genebank curators in the analysis of phenotypic and molecular marker data.

**a. Submodule one: Descriptor optimization and analysis of phenotypic diversity of genebanks**

The aim of this submodule is twofold: to select the phenotypic descriptors that are most useful in classifying a sample of genebank accessions into taxonomic groups or intraspecific groups predefined by the user, and to explore the diversity of a group of germplasm accessions with a set of phenotypic descriptors.

As mentioned above, genebanks not only register for each accession basic passport data, but may also collect characterization and evaluation descriptors which, along with passport data, are usually registered according to international standards (i.e., crop descriptors [20]). Crop descriptors can serve as a starting point to collect data that can be used in this submodule. These descriptors include characterization (e.g., botanical characteristics) and evaluation descriptors (e.g., yield, reaction to biotic or abiotic stress), which may differ in the way they are registered across multiple experiments or the degree in which they are affected by the environment. Usually, evaluation descriptors are highly influenced by the environment, so users should determine the quality of these data beforehand. For doing so, users should consider the methods used, experimental designs, statistical analyses applied, replicated trials performed over different years or locations, and the site and site environment descriptors that were registered. All these data will be useful to interpret the results coming from trials where evaluation descriptors were recorded. If this is the case, users should apply methods to analyze and validate these data to produce ready-to-use data, as done elsewhere [12].

The working example of this submodule analyzes a database that contains conventional morpho-agronomic traits (descriptors), and seed nutritional and biochemical traits, evaluated in a group of 1440 accessions, from the CIAT’s core collection of domesticated common bean (*Phaseolus vulgaris* L.). For this example, the domesticated accessions were classified into landraces of Mesoamerican, Andean or Colombian origin, according to their phaseolin type [21,22]. Andean landraces show phaseolins T, H, C, PA, CA, and TO, Mesoamerican landraces contain phaseolins S, M, Sb and Sd, and Colombian landraces show phaseolins B and CH. The database is organized in columns, with the ID of the accessions in the first column, followed by 44 descriptors. The descriptors include morphological traits from flower, seed, stem, and leaf, two phenological traits (Days to Flowering (DAF) and Days to Physiological Maturity (DAM), growth habit, phaseolin type, amylase inhibitor type, and nutritional compounds in the seed (concentration of micro and macronutrients and protein content). Additional descriptors are country of origin, municipality, and collection site. The database can be found at https://github.com/agrocompuepidemlab/GermVersity/releases/tag/v1.0.0. If users wish to reproduce the analyses with our example data, we advise them not to modify the additional descriptors because these are necessary for the biological interpretation of results.

The selection of descriptors was carried out using a machine learning approach [9] implemented in the package *randomForest* in R. The pipeline was as follows. Initially, Random Forests (RF) were used to perform accession classification based on phenotypic descriptors and Out Of Bag (OOB) accuracy of accession classification was calculated. For this, 70% of the data were used for training, and the remaining 30% for testing. The value of the Minimum Depth Distribution (MDD) and its relationship to the number of trees determined the selection of phenotypic descriptors of importance in the classification of accessions. Lower MDD values suggest more important traits in the classification. Furthermore, the confusion matrix enables the classification of accessions to be visualized. The confusion matrix can be examined in two ways: (i) the ability to distinguish between unrelated accessions and (ii) the sensitivity to detect phenotypic similarities across accessions of the same cluster [23].

Subsequently, with the descriptors selected in the previous stage, a multi-step approach was applied to a group of common bean accessions from CIAT’s core collection, with the aim of exploring their phenotypic diversity. For this, the number of phenotypic groups (*K*) was initially defined from a set of 26 clustering indices that support group selection by *K*-means. In our working example, the selected number of groups (*K*) was four, without limiting the clustering optimization for other case studies. For comparison purposes, users can visualize the clustering pattern of the accessions for two different *K* values. Finally, a principal component analysis was applied to display the relationships between each variable and the selected groups, as well as a hierarchical clustering analysis that allows the phenotypic similarity among groups of accessions to be visualized.

**b. Submodule two: analysis of the genetic stratification of a sample of germplasm accessions with SNP markers**

The aim of this submodule is to define the genetic structure of a sample of genebank accessions and calculate basic genetic diversity statistics. For the analyses of genetic data, the user must input a matrix of SNP data in VCF format. A VCF file can be built from sequencing data in *fastq* format, such as those derived from Illumina or other sequencing platforms, and a reference genome sequence. There are various tutorials available on how to obtain analysis-ready VCF files, to which the readers are referred (https://sourceforge.net/projects/ngsep/files/training/ManualNGSEP_v4.0.3.pdf/download). The SNP matrix should be previously filtered to minimize missing data (a maximum of 10%−20% missing data is recommended) and SNPs with rare variants [for example, a MAF (Minor Allele Frequency) of 5%]. The SNP file available for this demo (in VCF format) consists of 482 accessions of wild and domesticated Lima bean (*Phaseolus lunatus* L.) genotyped at 12,398 SNP loci with the reduced genomic scanning technique known as GBS (Genotyping-By-Sequencing) [24], using the restriction enzyme *ApeK*I. This VCF file has already been published, analyzed, and discussed elsewhere as source data [25].

For these samples there is associated metadata relevant to the analysis of genetic diversity, such as the biological status (wild or domesticated), country of origin and genepool for each accession. Previous studies have indicated that in this species there are four genepools for the wild accessions: two Mesoamerican (wild_MI, wild_MII) and two Andean (wild_AI, wild_AII); and two additional genepools for the domesticated accessions: Mesoamerican (DOM_MI) and Andean (Dom_AI) [25,26]. These two domesticated genepools originated from two independent domestication processes, one in Mesoamerica from the genepool MI in central-western Mexico, and the other from the wild genepool AI in the Andes of Ecuador-northern Peru. In this submodule we used this Lima bean dataset to show how to carry out analyses of genetic diversity to classify wild and domesticated accessions into genetic clusters using SNP data, and how to calculate basic diversity indexes for the observed genetic clusters. The VCF file and associated metadata can be found at https://github.com/agrocompuepidemlab/GermVersity/releases/tag/v1.0.0. Below we describe the process step-by-step.

The first step is to import the SNP matrix as a VCF file and the associated metadata in a text file. The latter is a tab-delimited text file with one row per accession, the first column with the taxa ID and the associated metadata in different columns. In the text file used for the working example, the second column is the biological status (wild or domesticated), the third column is the country of origin, and the fourth column is the genepool. From this last column, an object called “genepool” is created (this object is used in later analyses). When using his/her own dataset, the user can *a priori* define in the column called “genepool” the population to which each individual belongs. Accessions or individuals can be grouped according to the research questions and comparisons as required. If the population is not known, the analyses described below can help the user to define the population structure and optimum clustering. Then, the VCF file is converted (with the function *vcfR2genind* of the *vcfR* library) into a *genind* object (used by the package *adegenet* and other packages), indicating that the population assigned to each individual or accession is stored in the object “genepool”.

In the first analysis, the submodule calculates an Euclidean distance matrix between individuals on the basis of the observed allele frequencies among individuals. This distance is not a genetic distance *per se* (*i.e.*, *s.s.*) but a geometric distance since it does not assume any evolutionary model of molecular evolution. The function *nj* of the *ape* package [27] is used to build a tree topology using this distance matrix and the neighbor-joining algorithm [28]. The submodule also calculates a dendrogram, with bootstrap support of groups, from a *genlight* object. For this, a distance matrix is calculated, based on the proportion of loci that are different among individuals, and the UPGMA (Unweighted Pair Group Method with Arithmetic mean) algorithm is applied. The number of bootstrap replicates is set to 1,000. For this analysis, GermVersity applies the *aboot* function of the *poppr* package.

Sometimes the genetic unit of interest is the population and not the individual. For this reason, we implemented the construction of a genetic distance matrix among populations (or genepools) and the derived tree topology. Unlike geometric distances, genetic distances are based on a specific evolutionary model. Genetic distances can be seen as summary statistics because they take the whole dataset (for example, data from SNP loci) and summarize the genetic differentiation between samples (individuals or populations) in a value. One of the most used genetic distances is Nei’s [29], which measures the genetic distance among populations on the basis of their genetic identity, namely the proportion of alleles that are shared between pairs of populations. To calculate Nei’s genetic distances among populations we need to add the strata to the *genind* object (in this case based on the genepool) to define the populations. Then the sub-module uses this distance matrix to build a UPGMA tree and a neighbor-joining topology, applying 1,000 bootstrap permutations to get statistical support of the groups in the topologies. These analyses use the *aboot* function of the *poppr* package.

This submodule further explores the genetic structure of the sample with a Principal Component Analysis (PCA). PCA is a multivariate technique that summarizes the information provided by the genetic markers (in this case SNPs) into a few sets of components. The submodule applies PCA to a *genlight* object with the function *glPca* of the package *adegenet*. To identify genetic clusters, this submodule implements another multivariate approach known as Discriminant Analysis of Principal Components (DAPC) [30]. This approach is convenient when the interest is in describing the diversity among groups of individuals rather than within groups. This approach maximizes discriminant functions that better describe the differences among groups, while minimizing the differences within groups. However, to find the discriminant functions, the groups need to be known *a priori*, and in many cases this information is missing. To address this issue, the function *find.clusters* of the *adegenet* package is run to optimize the number of clusters by means of a PCA and the *K*-means algorithm. Then, it runs the function *dapc* to establish the relationships among clusters. In this submodule, the membership probabilities of each individual to each of *K* populations are calculated on the basis of the retained discriminant functions with the function *compoplot.dapc*.

After classifying the individuals into clusters or populations, the submodule calculates basic diversity indexes, first for the whole sample and per locus, and then for each cluster. For the analysis of the whole sample, it uses the function *summary* of the *adegenet* package to estimate the number of alleles per locus (NA), observed heterozygosity per locus (*H*_*O*_) and expected heterozygosity (*H*_*E*_) per locus. For SNP markers, NA might not be very useful because these loci are expected to be biallelic in populations (according to the infinite site mutational model). *H*_*O*_ is the proportion of heterozygous individuals that are observed in a locus. *H*_*E*_ is the heterozygosity expected to be observed in a locus assuming that the population (in this case the whole sample) is in Hardy-Weinberg Equilibrium (HWE) [31] at that particular locus. A significant difference among *H*_*O*_ and *H*_*E*_ means that the locus is not in HWE. Additionally, the submodule applies Bartlett tests to assess whether *H*_*E*_ is different from *H*_*O*_. As the number of loci, and therefore the number of multiple tests, increases, it is advised to correct the *p*-values for multiple comparisons (for example with the Bonferroni correction).

GermVersity also estimates genetic diversity indexes for each cluster. This is useful in finding out which cluster is the most or least diverse. To do this, it uses the function *basic.stats* of the program *hierfstat* to estimate observed heterozygosity (*H*_*O*_), mean gene diversities (*H*_*S*_) within populations, and the fixation index *F*_*IS*_. As stated before, a significant difference among *H*_*O*_ and *H*_*S*_ means that the population is not in HWE. As different populations may not be in HWE for various reasons, little comparison of diversity among populations can be made on the basis of *H*_*O*_. For this reason, *H*_*S*_ is preferred for the comparison of the genetic diversity between populations, as with *Hs* all populations are assumed to be in HWE. The fixation index *F*_*IS*_ measures the deviation to the assumption of HWE in the populations.

In order to determine how genetically divergent the populations are, GermVersity calculates the fixation index *F*_*ST*_, which measures the difference between *H*_*S*_ and the total genetic diversity expected in the whole population (*H*_*T*_). The higher the difference between *H*_*T*_ and *H*_*S*_, the higher the fixation index *F*_*ST*_, and therefore the higher the divergence among the populations. GermVersity utilizes the function *wc* of the program *hierfstat* to calculate *F*_*ST*_ (global and pairwise) according to Weir and Cockerham [32].

#### Module two: Spatial analysis and current and potential distribution of germplasm based on Ecological Niche Model (ENM) approach.

Ecological Niche Modeling (ENM), and Spatial Distribution Modeling (SDM), revolutionized the identification and use of novel genetic resources by analyzing the spatial distribution of wild and landrace germplasm accessions. Utilizing geographic and environmental data, ENM predicts where specific genetic resources can be found, assisting their targeted collection and conservation. This method not only supports conservation efforts but also bolsters pre-breeding programs by pinpointing ecological niches of accessions, thus helping breeders select parental lines. By integrating ENM, the GermVersity platform enhances genetic resource management, providing a detailed framework for conserving and utilizing genetic diversity. ENM’s strategic application improves the resilience and productivity of crops, contributing to sustainable agricultural development amidst environmental shifts. Among the many methodologies used for SDM, two widely implemented advanced machine learning techniques are Generalized Linear Modeling (GLM) and Maximum Entropy Modeling (MaxEnt). The GLM approach analyzes the relationship between the presence of species and environmental factors, identifying key areas for the conservation and use of genetic resources, while MaxEnt assumes that the most probable distribution of a species is the one that maximizes entropy, given the existing information on its presence and the environmental characteristics of those locations, and is particularly useful when absence data are scarce. Both methods are fundamental in informing decisions in pre-breeding and conservation programs, thanks to their ability to handle diverse variables and generate accurate predictive models.

The aim of this module is to explore ENM and SDM. The paradigm of SDM is used for wild and landrace accessions of germplasm collections. For SDM, two types of data are required: accurate georeferencing data of each accession and the environmental layers (rasters). The georeferencing data is a list in excel format with the column names: ‘Species’, ‘Longitude’, and ‘Latitude’. The format for geographical coordinates supported by GermVersity is decimal degrees. Users have two options for using the layer data. One is to use the 19 bioclimatic variables from WorldClim (https://www.worldclim.org/) directly from the repository at approximately 2.5 minutes (17.2 km^2^) resolution [33]. The other is to upload their own layers. A description of the 19 bioclimatic variables can be found at https://www.worldclim.org/data/bioclim.html. To streamline the acquisition of the environmental layers required for model execution, an update was implemented utilizing the *geodata* library, in conjunction with other tools such as the *terra* package. This integration was necessary due to recent updates in the ENMevaluate package, which now operates exclusively with spatial objects defined by the *terra* framework. This modification ensures compatibility across modules and facilitates a more efficient and reproducible modeling workflow.

GermVersity automatically downloads the different layers in raster format based on the coordinate of latitude and longitude at the collection points of accessions. As a first approximation we recommend directly using environmental layers in WorldClim (19 bioclimatic variables). However, we encourage users to implement their own environmental layers at a resolution greater than 30 seconds (approximately 1 km^2^) for improved precision in the output of spatial distribution modeling. It is advisable to carry out an *a priori* analysis of data curation, correlation, Variance Inflation Factor (VIF), or to use other analytical methods to reduce redundancy and pseudo-replication among environmental variables, with tools outside GermVersity such as those implemented in the program *Capfitogen3* (http://www.capfitogen.net/en/). Also, we suggest using inferred environmental indices for specific abiotic stresses rather than just using raw environmental variables, as implemented in ENVIREM (ENVIronmental Rasters for Ecological Modeling) resources (https://envirem.github.io/) [34]. The tutorial data for this module was based on Garcia et al. [25] and consists of the same accessions of Lima bean used in module one, submodule two. The original data of this module can be found at https://github.com/agrocompuepidemlab/GermVersity/releases/tag/v1.0.0.

This module offers two approaches for SDM, a classical Generalized Linear Model (GLM) function [35] and another algorithm using the popular Maximum Entropy (MaxEnt) method [36]. The GLM algorithm is implemented based on the R library *Caret* (Classification and Regression Training), widely used in data science. The first step is to extract the point values of the presence data from the user’s own environmental layers, or from those downloaded from WorldClim. Subsequently, a cleaning of missing values and selection of unique values by point of presence are performed. In parallel, pseudo-absences are captured with the function *raster::sampleRandom()*, avoiding sampling the same cell more than once. Next, presence and absence data are merged, having previously confirmed that both classes are balanced. Then the module proceeds to divide the data into training and testing sets at 80% and 20%, respectively. The resampling method chosen for class modeling is cross-validation with repetitions, for which 5 folds and 3 repetitions are fixed. Cross-validation with repetitions is a model evaluation technique that involves dividing data into groups (folds) to train and test the model multiple times in an iterative manner, repeating the process with different partitions to obtain a more accurate estimate of the model’s performance. By redoing the cross-validation several times and averaging the results, variability is reduced, and a more reliable evaluation of the model’s predictive ability is achieved. Subsequently, the training is carried out with the function *caret::train()* and the GLM algorithm modeling implemented in the *stats::glm()* protocol. The calculation of the accuracy of the model is made on the testing dataset to ultimately estimate the error of the model.

On the other hand, the MaxEnt is carried out by the R function *MaxNet* (https://github.com/mrmaxent/maxnet). The optimization of spatial distribution modeling via maximum entropy is implemented based on the R-package *ENMeval 2−0* [37]. Firstly, the user selects the number of background points as input in the function *ENMevaluate*. Spatial cross-validation with four folds is implemented as a resampling method to measure the performance of different predictive models. The predictive models are generated by combinations of various transformations (L = linear, Q = quadratic, H = hinge, P = product and T = threshold) of the original predictor variables (‘feature classes’). Then, the corrected Akaike Information Criterion (AICc) [38] is used as a reference metric to select the best performing model.

Finally, the prediction of spatial distribution is executed using the *raster:predict()* function for GLM and MaxEnt. For both methods, projections are transformed into dataframe format through the *sp::SpatialPixelsDataFrame()* function and visualized with high-quality graphics via the R *ggplot* library.

The ENM or SDM analytical pipelines are by definition more complex and rigorous than what is presented in this module, which includes multiple processes of basic elements for the correct use of these algorithms and its numerous applications [39,40]. For instance, in this exploratory approach, the optimization automations were configured with specific values and ranges for parameters of both GLM and MaxEnt. Examples include the number of background points, the number of repetitions and folds in cross-validation, feature classes, and the regularization multiplier. For this reason, we highly recommend that users of this module consult different protocols for the correct and responsible use of these approaches [41,42].

#### Module three: Genome-environment association tests.

Genome-Wide Association Study (GWAS) is a key technique in genetics that enables the identification of genetic variants associated with specific biological traits. In the context of genebanks, these studies are highly useful for allele mining. Firstly, GWAS facilitates the identification of genes of interest for crop breeding. These genes may become valuable tools for genetic enhancement, enabling the selection of superior varieties. Moreover, GWAS helps characterize genetic diversity in genebanks, providing essential information for the conservation of genetic resources and the selection of parental lines in breeding programs. Secondly, GWAS helps unravel the underlying genetic architecture of complex traits, namely those influenced by multiple genes and the environment. By identifying the genome regions and genes involved in the variation of these traits, a deeper understanding is gained, which can be applied to design more effective strategies for genetic breeding.

In some cases, the interest lies in understanding how populations have adapted to local environmental conditions. Identifying the genes that underlie adaptation processes relevant for crop breeding, *e.g.*, tolerance to drought, can be very useful in enhancing the value of germplasm collections. Genome-Environment Association (GEA) [43,44] among SNP markers and environmental variables or indices is an approach, based on the GWAS paradigm, that is implemented in this module. The utility of GEA studies lies in their ability to discern how environmental factors can modulate the influence of genes on the expression of complex traits.

Several analytical approaches have addressed the paradigm of GWAS analyses, the family of Mixed Linear Models (MLM) being one of the most commonly implemented tools in several species. Current MLM-based functions generally combine mixed linear models [45] with kinship and population structure as covariates to correct for false positives. However, new GWAS algorithms such as those implemented in GAPIT [46] and Latent Factor Mixed Model (LFMM) [47] have recently been developed in order to gain statistical power to assist in the detection of associated markers, to increase efficiency, and to decrease computational complexity [48].

**a. Submodule one: GWAS of genetic and environmental variables with GAPIT**

GAPIT is a genome association and prediction integrated tool freely available since 2011 [49] developed in the Zhiwu Zhang Laboratory (https://zzlab.net/GAPIT/) from Washington State University. Currently, Dr. Jiabo Wang is leading the newly-developed GAPIT version 3 that implements two new GWAS methods, FarmCPU [50] and BLINK [51]. These methods have shown to be more efficient in controlling false-positives due to confounding factors. In this submodule, the confounding effects of population structure are controlled by means of a PCA and a kinship matrix, both analyses based on genome-wide SNP markers. The novelty in both methods is in dividing the classic MLM algorithm into two parts that are used iteratively, a Random Effect Model (REM) and a Fixed Effect Model (FEM). BLINK replaces Restricted Maximum Likelihood (REML) in FarmCPU’s REM with Bayesian Information Criteria (BIC) in an FEM. López-Hernández & Cortés [52] explored the use of these tools to obtain GEAs in common bean using Genotyping-By-Sequencing (GBS) and customized environmental indices of heat stress.

For the GEA modeling, two types of data are required: the environmental data of each accession and the genomic data. The environmental data is a bio-variable or environmental index extracted from the specific collection site of each wild or landrace accession using georeferencing data. The environmental data can be provided as a list in excel format with the column names: ‘Taxa’, ‘Biovariable or Index 1’, ‘Biovariable or Index 2’… and ‘Biovariable or Index n’. The maximum number of environmental variables (columns) in the table are seven, apart from the ‘Taxa’ ID. The missing data is encoded as ‘NA’. The tutorial data for this submodule was based on accessions of wild Lima bean analyzed by García et al. [25] and the layers ‘bio1’ (average temperature), ‘bio12’ (average precipitation) and ‘bio3’ (isothermality) from the WorldClim platform, and the environmental indexes: ‘Annual PET’, ‘Aridity Index Thornthwaite’, ‘Climatic Moisture Index’ and ‘Continentality’ obtained from the ENVIREM resource.

For GAPIT, the genomic data is required in HapMap format (*hmp.txt*), initially used in the *International HapMap Project*. This format contains the genomic variation of SNP markers discovered and genotyped from Next Generation of Sequencing (NGS) methods such as GBS. A common format for GWAS analysis is the VCF format, which users can convert to *hmp.txt* format through popular resources such as the software Tassel 5.0 [53]. In this submodule, the genotypic data consists of 10,668 SNP loci, genotyped at 259 wild Lima bean accessions. MAF values are fixed at 5%. The environment and genomic data used in this module can be found at https://github.com/agrocompuepidemlab/GermVersity/releases/tag/v1.0.0.

The results generated by GAPIT are stored in the local folder ~ /GAPIT/. There are three tables for each variable of the phenotypic input and for each statistical approach (MLM. FarmCPU and BLINK) with information associated with: SNP identifier, chromosome, chromosome position, *p* value, MAF, *r*-square of model without SNP indexation, *r*-square of model per SNP, FDR-adjusted *p*-values and effect of the SNP. Also, for each trait, descriptive statistics as well as Manhattan and QQ-plots are provided. In addition, Manhattan and QQ-plots that show association results for all traits simultaneously are generated; therefore, we suggest the user input less than six variables at a time to be able to visualize these plots efficiently.

**b. Submodule two: Latent Factor Mixed Model (LFMM) analysis**

In this submodule, GermVersity implements a latent factor mixed model analysis (LFMM) to carry out GEA using. For this case study, we used the same genomic data as in submodule one, namely 259 accessions of wild Lima bean, that belong to four wild genepools (two Mesoamerican: MI and MII, and two Andean: AI and AII), and that have previously been genotyped at 10,668 SNP loci. Frichot et al. [54] implemented, in R’s *LEA* package, algorithms that combine linear regression with factor models to detect high correlations between allele frequencies, *e.g.*, from SNP loci, and environmental variables of interest, while controlling for the confounding effects of population stratification, demography or other factors. In this approach, population structure is modeled using latent factors (K) (that can be set with the number of ancestral populations in the sample), while the environmental variables are considered fixed effects.

Here we describe the steps for LFMM analysis. For the *LEA* package, the user should provide a genotypic matrix of SNP data, that can be in VCF format, but without missing data. This file can be obtained by using imputation methods such as those implemented in programs such as Tassel. The VCF file is then converted to *lfmm* format with the function *vcf2lfmm*. Then, environmental data is uploaded as a text file. This file should contain one row for each individual and one column for each environmental variable. This file does not contain any columns with identifiers for the individuals; therefore, the order of the individuals should be the same as in the VCF file. In this submodule, the environmental data for the case study consists of the variable bio10 (average temperature of the warmest quarter) because previous analyses have indicated that this variable is important for the ecological adaptation of wild Lima bean (unpublished data). The environmental data were obtained from the WorldClim database using the geographic coordinates of the collection site of each accession. The next step is to decide on the number of K latent factors to model population structure. For this, the strategy outlined above, in submodule two of module one, can be used to define the number of K populations in the sample. Since modeling population structure is not a straightforward matter, it is recommended to run the LFMM analysis with a range of *K* values. For example, for the wild Lima bean dataset, previous results have shown that the number of genepools is four, and in this case, we run the LFMM analyses for values of *K* from 3 to 10. After analyzing the results, we have chosen *K* = 8 as the best *K* value to model population substructure; therefore, in this submodule we only show the results for *K* = 8.

To run the LFMM analysis, the function *lfmm2* is first used to estimate the latent factors. This function returns an object that contains the following matrices: U, the estimated factors, and V, the loadings for all latent variables. Once the latent factors are estimated, association tests are performed with the function *lfmm2.test*, where population structure is accounted for, and *p*-values are adjusted by using genomic control. This function generates an object that contains the *p*-values of the association tests (one for each SNP locus), the scores of the Fisher’s tests, the effect size of the environmental variable on each SNP, and the genomic inflation factor (GIF).

After running the analyses for several values of *K*, the user should carefully inspect the results to decide whether confounding factors have been correctly controlled and choose the optimum value of *K*. For each *K*, the user can visualize the following: (a) the GIF, the best value of K should have a GIF value around 1, lower values are too conservative and higher values are too liberal; (b) a histogram of the *p*-values, the best value of K should show a flat histogram, with a peak around zero; and (c) a QQ-plot, the best value of K should have a QQ-plot where most of the observed *p*-values correspond to the expected *p*-values under the null hypothesis of no association, except for those *p*-values showing strong associations.

After the user has decided on the best value of K, candidate loci are identified. For this, a False Discovery Rate (FDR) control method is applied to convert *p*-values into *q*-values. Then, candidate loci under selection are identified according to an FDR threshold. In this example, a *q*-value < 0.1 is used as threshold. The last step is to combine the results of the association tests with the genomic positions of SNPs in a data frame. For this, the user should provide a text file with three columns, and the number of rows should equal the number of SNP loci analyzed. The first column, called “chrom”, is the chromosome number where each SNP is located; the second column, called “bp”, is the position of each SNP within the chromosome in base pairs (bp); and the third column, called “snp”, is the SNP ID. Finally, a Manhattan plot that shows the association results is built. The environment and genomic data used in this module can be found at https://github.com/agrocompuepidemlab/GermVersity/releases/tag/v1.0.0.

## Results

### Module one: Optimization and determination of relevance of descriptors and analysis of the phenotypic and genotypic diversity of genebanks

**a. Submodule one: optimization of morphological descriptors in genebanks and analysis of phenotypic diversity**

Not all conventional descriptors (*e.g.*, taxonomic descriptors) or all those acquired via high-performance phenotyping tools are informative. Therefore, optimization of these is necessary, not only to improve the characterization of diversity, but also to reduce the time and cost associated with their acquisition and computational analysis [9,20]. In the first part of this submodule, an example was developed using a database that contains conventional descriptors from the Bioversity International – CIAT’s Future Seeds genebank core collection of *P. vulgaris*.

**Fig 3:**
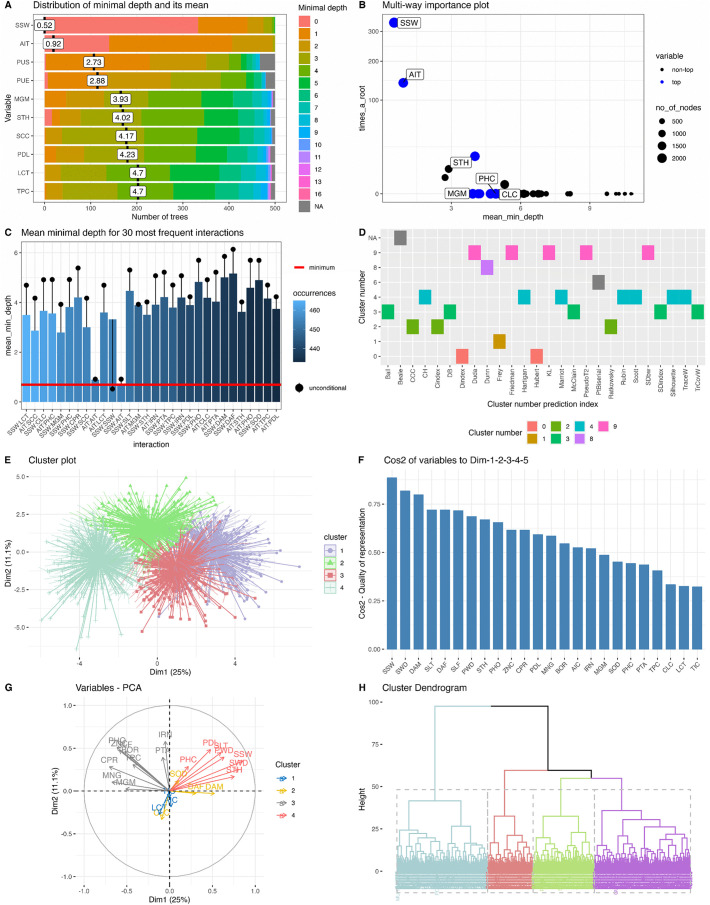
Results of module 1 – submodule 1: Selection of important descriptors using random forests and classification of accessions into phenotypic groups. A. Distribution of mean minimal depth and number of trees. B. Plot that relates the number of trees in which the root is split on a specific variable (times_a_root), the mean minimum depth, and the number of nodes. C. Mean minimum depth for the 30 most important pairwise variable interactions. The red line indicates the lowest mean minimum depth observed. D. A total of 26 clustering indices that aid in the selection of the number of phenotypic clusters of accessions. E. *K*-means clustering based on phenotypic traits for *K* = 4. F. Contribution of phenotypic variables in principal component analysis. G. Eigenvectors of the relationships among phenotypic descriptors and phenotypic clusters. H. Hierarchical clustering of accessions based on phenotypic traits. SSW:100 seed weight, AIT: Amylase inhibitor type, PUS: adaxial pubescence of leaf, PUE: abaxial pubescence of leaf, MGM: magnesium content, STH: seed thickness, SCC: seed coat color, PDL: pod length, LCT: lectin content, TPC: total protein content.

The implemented analyses allowed the optimization of descriptors based on the distinction they may offer of common bean accessions previously classified in genepools (Mesoamerican, Andean and Colombian) based on phaseolin type [22]. GermVersity generates two types of outputs during descriptor optimization. The first output is related to model performance that can be evaluated with the confusion matrix and the Kappa index. In the working example, our analysis shows that the classification of bean genepools has an accuracy of 0.84 and a Kappa index of 0.72, showing that the performance of the model is very good (see below). The second output is related to the selection of important descriptors and the classification of accessions into phenotypic groups by applying *K*-means clustering, principal component analysis, and hierarchical clustering (Fig 3).

GermVersity produces three graphs that enable the comparison of classification precision of descriptors; these consist of a plot depicting the distribution of Mean Minimal Depth (MMD) and number of trees (Fig 3A), a plot that relates the number of trees in which the root is split on a specific variable (times_a_root), the MMD and the number of nodes for the most discriminant descriptors (Fig 3B), and the MMD for the 30 most important pairwise variable interactions (Fig 3C). For our case study, Fig A shows the ten descriptors that classify the three gene pools to the greatest extent. Of these, SSW (100 seed weight) and AIT (amylase inhibitor type) are among the most relevant since they show low MMD values (a lower MMD means that the first node for which a descriptor is used to split the accessions is closer to the root node). The most essential descriptor was SSW, which showed the highest times_a_root, of approximately 320, and the smallest MMD (0.52) (Figs 3A and 3B). Finally, the results show the MMD of the 30 pairwise variable interactions that contribute the most to the classification of common bean genepools (Fig 3C). The six most frequent interactions often include seed (100 seed weight-SSW, amylase inhibitor type-AIT, protein content-TPC, sodium content-SOD, phosphorous content-PHO, and seed thickness-STH), pod (pod length-PDL) and phenological traits (days to flowering) (DAF); therefore, these interactions may serve as tools to characterize the common bean germplasm. These results are in accordance with Singh et al. [21] because these authors also found that seed size and days to flowering are relevant to the classification of common bean accessions into genepools. In summary, this analysis aids in identifying the most important descriptors as well as those with the greatest interaction with other descriptors. In this case, SSW and AIT, when combined with other descriptors, contributes to the accuracy of the classification model of common bean. We advise users to evaluate the performance of the model implemented in GermVersity based on their expertise with the data and the species group they are analyzing.

In addition to descriptor optimization, GermVersity integrates a set of tools that allow the phenotypic variation of bean accessions to be understood using *K*-means, Principal Component Analysis (PCA), and hierarchical clustering. To define the number of clusters, GermVersity calculates 26 clustering indices that the user can examine. For the working example, seven indices suggest an optimum number of clusters of four (*K* = 4) (Fig 3D) and not *K* = 3 corresponding to the three genepools. The interpretation of these clusters should be done in connection with the phenotypic descriptors assessed and, if possible, the evolutionary history of the species. In the case study, of the four clusters suggested by *K*-means, two clusters (*K*1 and *K*3) were mainly composed of Andean landraces and the other two clusters (*K*2 and *K*4) were mainly composed of Mesoamerican landraces. Regarding the Colombian landraces, 86% of them grouped with Mesoamerican landraces in clusters *K*2 and *K*4, and some few (14%) grouped with Andean landraces in clusters *K*1 and *K*3. Since previous authors have shown that Colombia is a meeting place for Mesoamerican and Andean landraces [22], where introgressions among genepools may take place, this biological phenomenon could explain these results.

GermVersity also allows visualization of the results for *K* values defined by the user (Fig 3E, for the working example *K* = 4). The results of the PCA show that the descriptors with the highest square cosine values include 100-seed weight, seed width (SWD) and days to maturity, among others (Fig 3F). Fig 3G displays the relationships between descriptors and the four phenotypic clusters. Finally, for the working example, hierarchical clustering revealed four major groups, pinpointing the accessions that belong to each of the clusters (Fig 3H). The methodology allows the degree of similarity among accessions to be determined. This in turn enables the identification of functional groups in genetic resources, as well as the inference of phenotypic redundancy among accessions, which may be checked and verified via genetic analysis as part of the additional sub-modules.

**b. Submodule two: analysis of genetic diversity in genebank accessions**

**Fig 4:**
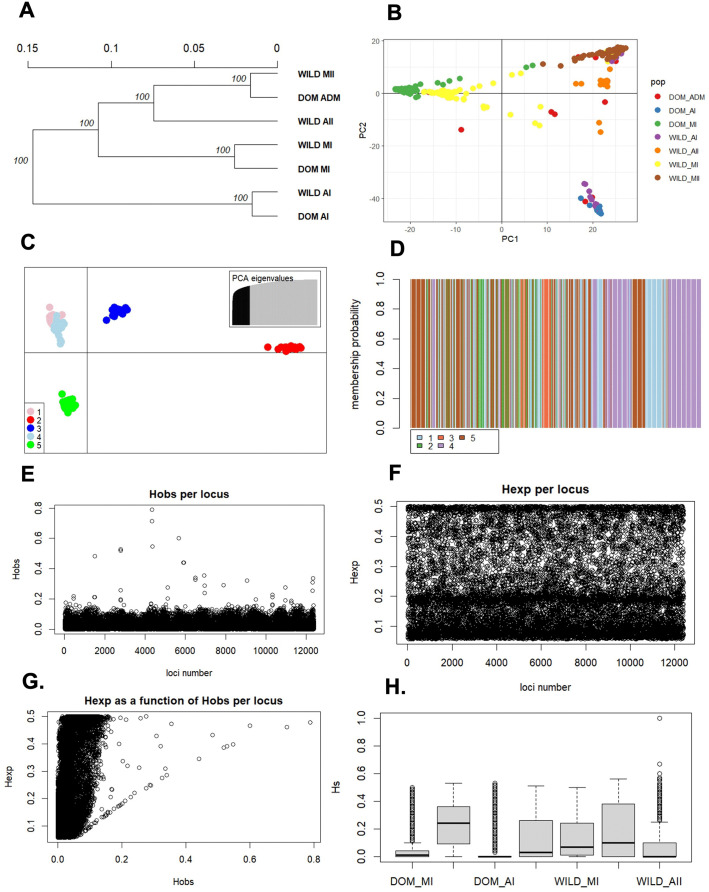
Results of module 1- submodule 2: Graphical results of the genetic diversity analyses. A. The user can estimate Nei’s genetic distances among predefined populations and visualize the genetic relationships through a UPGMA dendrogram. If the number of populations in the sample is not known, the user can apply: **B.** a principal component analysis, and **C.** a Discriminant Analysis of Principal Components (DAPC) and, based on those results, define the number of populations. D. A population can also be assigned to each individual according to membership probabilities as visualized in a bar plot. Regarding genetic diversity statistics, the user can visualize plots of: **E.** observed heterozygosity (Ho) per locus, **F.** expected heterozygosity per locus (Hexp), **G**. the comparison among Ho and Hexp per locus, and **H**. the magnitude of the genetic diversity in the populations.

This submodule carries out basic genetic structure analyses of germplasm accessions with SNP markers. As an example, this submodule worked with a sample of wild and domesticated accessions of Lima bean with a clear genetic structure defined by genepools in previous studies [25]. The first analysis consisted of exploring the genetic relationships among individuals by calculating an Euclidean distance matrix. The user can visualize a NJ tree and a UPGMA dendrogram, where individuals are colored according to the genepool (figure not shown). For this dataset, the clustering pattern corresponds to the genepools defined previously for this species. Then, the genetic relationships among populations (in this case the genepools) were also explored with a Nei’s genetic distance matrix. The relationships among populations can be visualized in an NJ tree and a UPGMA dendrogram (Fig 4A).

In many cases, the genetic structure of the sample is not known, therefore this module implements a PCA and a DAPC analysis to help the user infer the number of populations in the sample. From these analyses, the user can visualize a basic PCA scatter plot (Fig 4B). For the example dataset, the first principal component splits the accessions of the MI genepool from the rest of accessions (MII, AII and AI), while the second component separates the AI accessions from the MII and AII genepools. Also, accessions from genepool AI appear as more distantly related to the other genepools, a pattern that was also observed in the NJ tree and UPGMA dendrogram.

In DAPC, clusters are inferred with the function *find.clusters* and then individuals are assigned to the inferred clusters with the *dapc* function. The function *find.clusters* asks interactively for the number of PCs to be kept (we chose 500, more than the maximum for this dataset), as well as the number of clusters to be retained (we chose *K* = 5 according to the BIC values and previous biological knowledge). Choosing the “right” number of clusters is a complex issue, the BIC graph being just a guide. The researcher should explore different numbers of clusters and choose the number that makes most sense from a biological point of view. The function *dapc* asks again for the number of PCs and discriminant functions to be retained. For this example, only 100 PCs were retained, to avoid overfitting, and all four discriminant functions were kept. As a result of this analysis, the user can visualize a basic scatter plot of DAPC (Fig 4C) and can inspect, in a table, the correspondence among the groups defined by *find.clusters* and the previous assignment of accessions into genepools. For this example, the correspondence among the *K* clusters (5 groups) and the predefined genepools is acceptable (table not shown). The user can also inspect, in a table and a barplot (Fig 4D), the cluster each accession was assigned to, and in another table, the assignment membership probabilities per individual to each one of the *K* populations on the basis of the retained discriminant functions.

The last set of analyses of this submodule calculated basic diversity statistics per locus and population. Regarding basic diversity statistics per locus, the user can inspect a plot that shows the observed and expected heterozygosity per locus (H_O_ and H_E_, respectively) (Figs 4E, 4F). As expected for a selfing species such as Lima bean, in most loci H_O_ is low, in general much lower than 0.2, and a great variation in H_E_ was observed, with a maximum value of 0.5, since all SNP markers analyzed were biallelic. Also, a plot that shows how H_E_ is related to H_O_ can be visualized (Fig 4G). For loci in HWE, H_E_ will be similar to H_O_. In the plot it is observed that for most loci, H_E_ is higher than H_O_, as expected for selfing species. Regarding basic diversity statistics per cluster (or population), the average genetic diversity (H_S_) shows that populations are different (Fig 4H), with the highest values observed for the domesticated accessions classified as admixed (meaning than that resulted from gene flow among genepools), followed by the Andean wild genepool AI. The submodule also estimates F_IS_ values within populations, calculated as (H_S_-Ho)/H_S_ (Ho: observed heterozygosity, Hs: genetic diversity). For the example dataset, F_IS_ values are positive for all populations and some of them are close to 1 (results not shown in Fig 4), as expected for a predominantly autogamous species such as Lima bean.

Finally, this submodule calculated the genetic divergence among populations. The user can inspect a useful plot that shows the overall difference between H_S_ (average genetic diversity of populations) and H_T_ (genetic diversity of the total sample) (not shown in Fig 4). For the example dataset, the score for H_S_ was much lower than H_T_, which suggests a certain degree of differentiation among populations, as measured by F_ST_ [F_ST_ = (H_T_-H_S_)/H_T_]. In this analysis, genetic differentiation among genepools was high (*F*_*ST* _= 0.59), as was deviation from HWE, with a large heterozygote deficit (*F*_*IS* _= 0.69), a consequence of the Wahlund effect. Finally, a matrix of pairwise values of F_ST_ among populations was also calculated (results not shown in Fig 4). The highest F_ST_ values (highest genetic differentiation) were observed among genepools Dom_MI *vs.* Dom_AI, Dom_MI *vs.* Wild_AII, and Dom_AI *vs.* wild_AII. The lowest genetic differentiation was observed among genepools Dom_ADM *vs.* wild MII, Wild_AI *vs.* Dom_AI (Andean wild ancestor and its domesticated counterpart), and Wild_MI *vs.* Dom_MI (Mesoamerican wild ancestor and its domesticated counterpart).

### Module two: Spatial analysis and current and potential distribution of germplasm based on Ecological Niche Model (ENM) approach

The data implemented as case study for the modeling of the spatial distribution of species is the legume Lima bean. Our dataset recovered the georeferencing of accessions located from Mesoamerica to Argentina, analyzed by García et al. [25]. Data was previously cleaned for missing data and accessions outside the mainland. Meanwhile, access to the environmental layers was provided through the WorldClim platform, at a resolution of 2.5 minutes (~4.7 km) for the 19 historical annual bio-variables of WorldClim.

**Fig 5:**
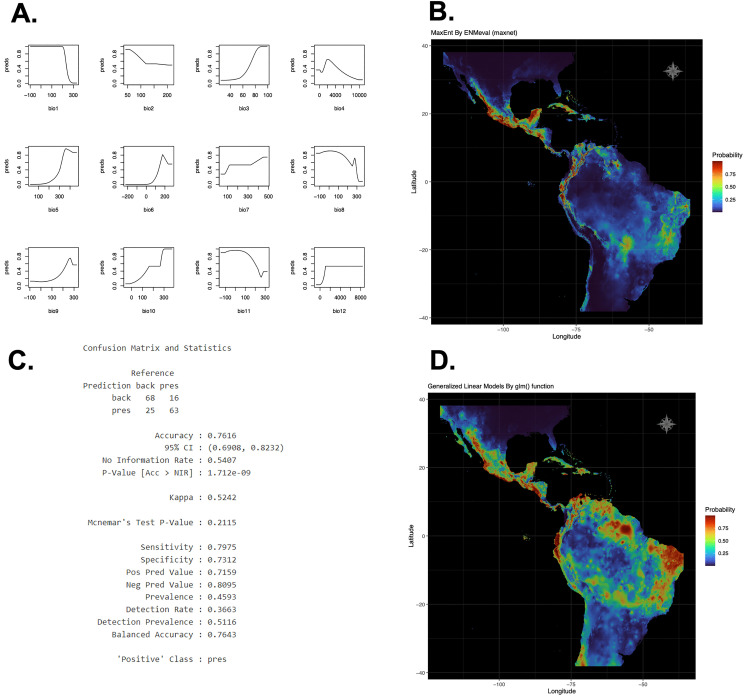
Results of module 2. Spatial Distribution Modeling (SDM) by Maximum Entropy algorithm and a Generalized Linear Model (GLM). A. The predictive behavior plots of the model for each WorldClim bio-variable. B. Spatial distribution modeling plot using the maximum entropy algorithm. C. Evaluation metrics of testing set using the GLM function (*e.g.*, accuracy, Kappa Index). D. Spatial distribution modeling plot using a GLM.

After loading the environmental layers, the maximum entropy optimization function, which collected different metrics for each combination of several transformations of the original variables, was automatically executed. Then, the results from each model were organized by feature classes (L, LQ, LQH, H) and subsequently by the regularization multiplier (RM). The model featuring a combination of linear, quadratic, and hinge transformations (LQH) achieved the lowest corrected Akaike Information Criterion (AICc) value of 9883.854, as shown in S1 Fig. The predictive behavior plots of the model for each WorldClim bio-variable are shown in Fig 5A. Subsequently, the spatial distribution map was plotted from the class probability obtained in the prediction made with the model for each cell of the territory (Fig 5B).

On the other hand, the Generalized Linear Model (GLM) randomly distributed 80% of the data for training, and the remaining 20% for testing. Then, a balanced sampling of pseudo-absence classes and points of presence of 53% and 47%, respectively, was implemented for the training data, and of 54% and 46% for the testing data. The training dataset was further explored by cross-validation with 15 iterations of the GLM, for which an average accuracy of 78.27% and Kappa index of 0.5651 were obtained. From the trained model, the prediction was made with the testing set that obtained an accuracy of 76.16% (CI95: [69.08%, 82.32%]) and a Kappa index of 0.5242 (Fig 5C). Finally, the spatial distribution map was plotted from the class probability obtained in the prediction with the best model for each cell of the study area (Fig 5D).

In our working example, the spatial niche modeling map by maximum entropy of *P. lunatus* suggested a distribution with high probability of presence from the Pacific coastal plain, the western Sierra Madre and the Yucatan Peninsula in Mexico, through to the Central American mountain range, the Andean Mountain ranges of Colombia and Ecuador and the Andean region of northern Peru in the Cajamarca region. On the other hand, the GLM approach suggests the same regions of Latin America mentioned above, but with an expansion into the tropical rainforest and thorny scrub areas of Brazil. This shows the preference of this bean species for regions with low-moderate altitude and climates classified according to the Köppen-Geiger classification as tropical with summer and monsoon rains, as well as tropical savanna and semi-arid climates.

### Module three: Genome-Environment Associations (GEA)

**a. Submodule one: Genome-wide Association Study (GWAS)**

**Fig 6:**
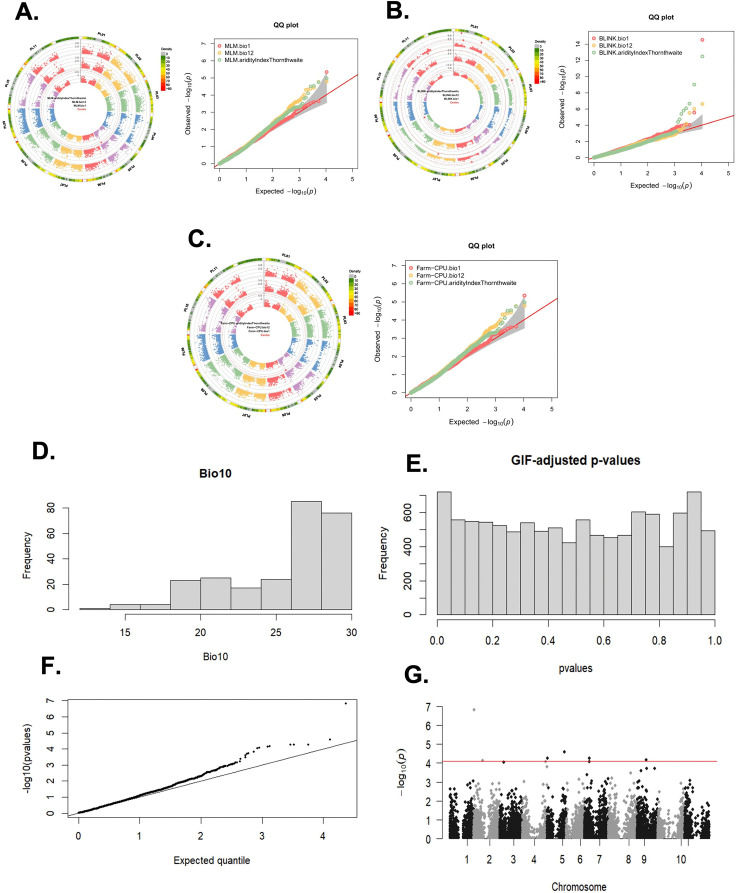
Results of module 3. Submodule 1. Genome-wide Association Study (GWAS) using three approaches: A. MLM (Mixed Linear Model), B. FarmCPU (Fixed and random model Circulating Probability Unification) and C. BLINK (Bayesian-information and Linkage-disequilibrium Iteratively Nested Keyway). Submodule 2: Graphical representation of the results of the LFMM analyses. D. Histogram of a bioclimatic variable (bio10 – average temperature of the warmest quarter). Histograms of any other variable can also be requested. For any value of latent factors, the user can also visualize: **E.** a histogram of the GIF-corrected *p*-values, **F.** a QQ-plot, **G.** a Manhattan plot with the association results.

The input of phenotypic data was consolidated from two raw environmental variables and an abiotic stress index. The two raw variables were obtained at a maximum resolution of 30 seconds (1 km) from WorldClim for mean temperature (bio1) and mean precipitation (bio12). Also, the environmental drought stress index ‘aridityIndexThornthwaite’ was accessed from the ENVIREM repository at the same resolution of 30 seconds. These three bio-variables were tabulated following the format mentioned in the materials and methods section with the identifier per individual in the column “Taxa”. On the other hand, the VCF file was converted by the TASSEL program [53] to the “.hmp.txt” format where all “Taxa” codes were validated with the phenotypic format. The graphical results that are previewed in this submodule are a Manhattan Plot and a QQ-plot for each analytical approach (MLM, FarmCPU and BLINK), and for all variables simultaneously (Figs 6A-6C).

The results for average temperature (bio1) show that in two approaches (MLM and FarmCPU) one significant association was detected (Figs 6A and 6C). The associated SNP is SPL05_34005730 located at position 34,005,730 of chromosome 5. On the other hand, the BLINK approach for bio1 detected two associated SNPs (Fig 6B). One of these SNPs is again SPL05_34005730, and a new SNP on chromosome 8 (SPL08_56203978). Also, as expected for this novel approach that better controls false positive and negative rates, significant associations were obtained for the other two variables, with three SNPs for average precipitation (bio12) and six SNPs for aridityIndexThornthwaite drought stress index (Fig 6B).

### Submodule two: Latent Factor Mixed Model (LFMM)

In this submodule, a LFMM analysis was run to detect SNPs highly correlated with an environmental variable. As a working example, this submodule analyzed 10,668 SNPs genotyped in a sample of 259 wild Lima bean accessions and correlated these SNPs with the variable bio10 (average temperature of warmest quarter) (Fig 6D). Before running the correlation tests, the number of latent factors was estimated. This number can be initially approximated by the number of *K* populations. In this example, the number of latent variables was set to 8. For any given value of *K*, the user can inspect the following results of the correlation tests: the Genomic Inflation Factor (GIF), a histogram of GIF-corrected *p*-values (Fig 6E) and a QQ-plot (Fig 6F), to decide whether confounding factors have been correctly controlled. Based on these results, the user can choose the best value of K to identify candidate loci. In the current working example, with *K* = 8, and a *q*-value threshold of 0.1, ten SNPs were detected as highly correlated with the environmental variable (see the Manhattan plot in Fig 6G). The same information is also summarized in a table where the following information is registered for each of the SNPs: GIF-calibrated *p*-value, *q*-value and the adjusted r-square. These results, and those of the GAPIT submodule, can be used in downstream analyses aiming to identify candidate genes for adaptation.

## Discussion

The need to preserve biodiversity and secure genetic resources for future generations has led to the establishment of genebanks worldwide [1–3]. In recent years, advancements in phenotypic and genetic characterization of germplasm banks have produced massive information associated with multiple accessions [55]. In parallel to this, it is necessary to develop high-throughput phenotyping and genotyping screening techniques, as well as tools for data integration and analysis [9]. If these tools are designed as end-user platforms, they can be accessed by users without advanced programming skills. In this sense, technologies have paved the way for the development of digital platforms as innovative tools for managing and analyzing biodiversity and germplasm data.

In view of the above, the development of a digital platform for the multi-analysis of phenotypic and genetic diversity in germplasm banks is a significant advancement in the field of plant pre-breeding and sustainable use of biodiversity. GermVersity platform, designed in Shiny, provides a user-friendly interface for researchers to access and analyze data from various datasets. This tool integrates multiple analytical approaches that can be applied to data associated with accessions from genebanks. The three modules seek to achieve integrated management of information, and the identification of potential functionality of existing diversity from multiple accessions of genebanks with available phenotypic and genetic data. The potential uses of GermVersity include but are not limited to: (1) optimization of discriminant morphological characters, (2) exploration of genetic diversity through phenotypic and SNP data analyses and identification of genetic stratification, (3) identification of potential distributions of plant species for conservation and prioritization of sampling areas, (4) reconstruction of the genomic architecture of phenotypic and adaptive variation, and (5) correlation among genetic and environmental variables.

The platform is continuously improved and has already been used by researchers to conduct a number of important studies as part of a *beta* unreleased version. For example, the platform was used to predict the potential distribution of the *Phaseolus* genus across the Americas using an ecological niche model approach, and to identify genes associated with specific environmental conditions of regions prone to drought and high temperatures (unpublished data). Based on the specific case study, the results can potentially be used to inform bean breeding programs, which will help to guarantee sustained bean production by improving the crop’s strategies to adapt to and mitigate climate change effects. The platform is a valuable resource for researchers working in plant breeding and ecology. It provides a user-friendly interface for accessing, analyzing, understanding, and mining data.

Currently available in version 3.0, GermVersity offers a suite of analytical tools tailored to meet the needs of academic users, genebank managers, researchers, and developers. In the era of data science and artificial intelligence advancement, GermVersity stands at the forefront, leveraging cutting-edge technologies for enhanced efficiency and accuracy in genebank management. In its current version, GermVersity already presents numerous opportunities for improving, updating, and integrating new analyses. We encourage academic users, developers, and the broader public to actively engage with GermVersity, to provide feedback, and to contribute to its ongoing enhancement. By collaborating on GermVersity’s development, we can collectively harness the vast potential stored in genebanks, driving innovation in agriculture, and ensuring the sustainable utilization of genetic resources for generations to come.

Finally, a comparison with current platforms positions GermVersity as a notably innovative user-friendly platform with a comprehensive, multi-module approach to data analysis, which contrasts favorably with the often steep learning curve and time-consuming nature of manual command processes of other platforms (Table 1). Compared to standalone tools such as TASSEL [53], which requires Java-based setup and offers limited cross-platform stability, or MLMM, which lacks GUI support and demands custom scripting, this Shiny-based system provides a reproducible, integrated, and fully R-native environment. Similarly, widely used SDM tools like MaxEnt GUI [36], openModeller [56], and SDMtoolbox [57] offer either limited automation, fragmented workflows, or steep learning curves—often requiring separate environments, GIS knowledge, or proprietary software like ArcGIS. On the GWAS side, command-line tools such as PLINK [58], EMMAX [59], FaST-LMM [60], GCTA [61], and Eigenstrat [62] are powerful but demand high technical proficiency, data preprocessing expertise, and often lack an integrated visualization layer. By contrast, the Shiny-based platform unifies state-of-the-art SDM and GWAS methodologies within a user-friendly and guided interface, enabling high-level analytics without requiring deep programming skills or extensive pipeline configuration. Furthermore, it is free, open-source, and installation-friendly, removing licensing and infrastructure barriers for researchers in low-resource contexts and offering a centralized solution for biodiversity and germplasm data analysis.

**Table 1:** Comparative analysis of GermVersity with other tools and manual command processes in genebank data.

Feature	GermVersity	Other tools/software [36,54,56–62]	Manual commands
User interface	User-friendly, in-house data analysis, easy installation	Varies; some command-line, some with GUI	Not applicable
Data analysis capabilities	Comprehensive, multi-module	May specialize in specific analyses	High flexibility
Data types supported	Quantitative, qualitative, continuous, discrete	Depends on the tool; some may have limitations	High flexibility
Integration capabilities	Robust integration with various data formats	Varies; some require extensive pre-processing	Requires in-depth knowledge
Cost	Free	Ranges from free to fee-based	Free
Accessibility	Easy installation and access	May require installation or subscriptions	Requires scripting knowledge
Customization	Limited customization options	Highly customizable depending on the tool	Fully customizable
Learning Curve	Relatively low	Varies; some tools have a steep learning curve	Steep
Limitations	Default workflows The platform is supported by functions previously defined in R, so continuous maintenance is required.	It varies; may not support all types of data, licenses required, high costs, restricted number of users, little cooperation with the academic community	Time-consuming, error-prone
Potential Developments	Expansion to include more data types, improved customization, software independence, collaboration opportunities with global developer communities	Varies; ongoing development common	Not applicable

Unlike manual commands, which require a deep knowledge of scripting and are prone to error, GermVersity provides an in-house, easy-to-navigate interface that facilitates immediate and broad access, especially for those less versed in programming languages. While GermVersity stands out for its robust integration capabilities with various data formats and its free accessibility, it does have limitations, such as less scope for customization compared to other tools and manual methods, which offer fully customizable environments to fit more specific research needs. Furthermore, GermVersity’s reliance on predefined workflows could be a constraint for advanced users seeking a high degree of analytical flexibility. In contrast, other tools and software might provide more specialized analyses, yet they often come with their own set of challenges, including the potential requirement for extensive pre-processing of data, possible license costs, and occasionally, limited cooperation with the academic community. Looking forward, the development potential of GermVersity includes expanding its data types to encompass more diverse datasets and enhancing customization and interoperability features. This would ensure GermVersity not only maintains its user-friendly appeal, but also grows in sophistication and adaptability, progressively aligning itself with the needs of global diversity conservation efforts.

This study introduces GermVersity 1.0, a prototype platform developed in R for the integration of genotypic, phenotypic, and spatial analyses in germplasm research. While R offers flexibility and analytical power, one of its main limitations lies in the obsolescence and lack of ongoing support for several packages, which are no longer available on CRAN. Recognizing this constraint, our future development efforts will focus on transitioning GermVersity into a standalone executable application (.exe). This will enhance the platform’s long-term stability, usability, and accessibility, especially for users with limited programming experience. Such a transformation will also reduce dependency on external package repositories and ensure consistent user experience. Meanwhile, we strongly encourage users to follow the detailed instructions provided in the user manual particularly regarding the installation and use of deprecated libraries which can be found in the official repository: https://github.com/agrocompuepidemlab/GermVersity/releases/tag/v1.0.0.

## Conclusions

The Shiny-based digital GermVersity platform represents a significant advancement in the analysis of phenotypic and genetic diversity within germplasm banks. In each module, the platform provides a comprehensive framework for data exploration and decision-making in pre-breeding programs and genebank management. Continued development of the platform, addressing limitations and integrating emerging technologies, will further enhance its capabilities, contribute to sustainable agriculture, and aid the conservation and utilization of genetic resources.

## Supporting information

S1 Fig The corrected Akaike Information Criterion (AICc) from the optimization of spatial distribution modeling via maximum entropy. (TIF)
